# National Trends in Pulmonary Embolism Visit in United State Emergency Departments and Associated Costs (2006–2018)

**DOI:** 10.1155/emmi/6610196

**Published:** 2025-01-20

**Authors:** Ahmad Aalam, Diyaa Bokhary, Awad Alsabban, Ahmad Bakhribah

**Affiliations:** ^1^Department of Emergency Medicine, King Abdulaziz University, Jeddah, Saudi Arabia; ^2^Department of Internal Medicine, Taif University College of Medicine, Taif, Saudi Arabia

## Abstract

**Introduction:** Pulmonary embolism (PE) poses substantial morbidity and mortality risks, necessitating timely and accurate management in emergency departments (EDs).

**Objectives:** This study explores the trends in PE presentations to US EDs from 2006 to 2018 and assesses the impact of different factors on management and cost.

**Methodology:** This is a retrospective descriptive study conducted using the US Healthcare Cost and Utilization Project (HCUP) PE ED visits database. Data on ED visits, dispositions, and related costs were collected and analyzed.

**Results:** From 2006 to 2018 there were more than 2 million PE ED visits in the US. There was an increase in visits per 100,000 persons from 42.17 in 2006–2008 to 64.27 in 2016–2018 (*p* value < 0.001). The proportion of uninsured patients declined from 5.07% to 4.70%, and the percentage of Medicaid-insured patients increased. There was a decrease in the admission rates from 92.47% to 75.97% in 2016–2018 (*p* value < 0.001). The mean cost per admitted patient increased from $32,794 to $47,344 in 2016–2018 (*p* value < 0.001).

**Conclusion:** From 2006 to 2018, PE ED visits in the US increased with a noticeable decrease in admission rates and length of stay, likely secondary to advancement in diagnostic and therapeutic modalities like computed tomography pulmonary angiography and novel oral anticoagulants. However, the observed rising healthcare costs pose challenges to sustainable management. Further research studies are needed to address cost-effective strategies.

## 1. Introduction

Pulmonary embolism (PE) is a critical pulmonary vascular disorder characterized by the obstruction of pulmonary arteries due to thromboembolic material, leading to compromised blood flow and oxygenation, thereby posing significant morbidity and mortality risks [1, 2]. As the third-leading cause of cardiovascular mortality following coronary artery disease and stroke, timely and accurate management in emergency departments (ED) is paramount for patient outcomes and resource optimization [3].

In recent years, PE research has garnered considerable attention owing to the advancements in diagnostic technologies and therapeutic approaches. Notably, the adoption of multidetector 64-slice computed tomography pulmonary angiography (CTPA) has revolutionized diagnostic accuracy, paralleled by a notable increase in CT utilization among ED presentations suggestive of PE [4, 5]. These advancements not only improve the accuracy of diagnosis, but also enable the detection of smaller emboli, including subsegmental and subclinical emboli.

Concurrently, the landscape of anticoagulation therapy has also witnessed an evolution, with the introduction of novel oral anticoagulants (NOACs) in 2010 offering an alternative to conventional regimens [6]. Landmark trials such as the EINSTEIN-PE trial (2012) and AMPLIFY trial (2013) have examined the efficacy and safety profiles of rivaroxaban and apixaban, respectively, reshaping therapeutic paradigms in PE management [7, 8].

Moreover, considerable research effort has been directed towards refining risk stratification and clinical decision rules for PE. The validation of scoring systems such as the Wells score (2004), the revised Geneva score (2007), the PERC score (2008), and the introduction of the YEARS algorithm (2017) aimed to optimize patient outcomes and resource utilization in ED settings [9].

This study sheds light on the trends of patients presented with PE to US EDs from 2006 to 2018. Our objective is to analyze how advancements in diagnostic tools, therapeutic modalities, and healthcare policies have influenced the management and costs associated with PE over time.

## 2. Methods

### 2.1. Study Design

This is a retrospective descriptive study of PE ED visits, dispositions, and related costs. Data were sourced from the Healthcare Cost and Utilization Project (HCUP), specifically the National Emergency Department Sample (NEDS) and National Inpatient Sample (NIS) databases, for the period between 2006 and 2018. Currently HCUP cover almost 97 percent of US population, therefore, datasets provide weighted national estimates based on a sample of US hospitals [10].

### 2.2. Data Collection

Data retrieval was performed for all ED visits and hospital discharges for all-cause newly diagnosed PE using the International Classification of Diseases (ICD) coding system. The ICD ninth revision Clinical Modification (ICD-9-CM) codes covered the years 2006–2014, while the ICD tenth revision Clinical Modification (ICD-10-CM) codes were used for the years 2016–2018. The transitional year 2015 was excluded because of the shift from ICD-9-CM coding system to the newer ICD-10-CM coding system. The online guide provided by HCUP was used to identify specific codes relevant to the diagnosis of PE. Subsequently, the data were sub-grouped into four distinct periods of three years each (2006–2008, 2009–2011, 2012–2014, and 2016–2018) to facilitate temporal analysis.

Data extracted included age, gender, insurance status, income, ED disposition, hospital geographics, admission length of stay (LOS), outcome either death or discharge, cost per admission, and aggregate cost. HCUP delineates cost utilization as the cost-to-charge ratio derived from hospital accounting reports sourced from the Centers for Medicare and Medicaid Services (CMS), adjusting dollar figures to account for inflation using the Consumer Price Index (CPI) and are expressed in 2018 US dollars.

### 2.3. Data Analysis

MedCalc statistical software version 22.016 was used for statistical analysis. Chi-square test was used for categorical variables, while independent *t*-test was used for continuous variables. A *p* value of < 0.05 was considered statistically significant. Since the HCUP datasets aggregate data, logistic regression was not feasible, and limitations associated with aggregate-level analysis have been acknowledged.

## 3. Results

### 3.1. Demographics

Over the study period, more than 2 million US PE ED visits were reported. We found a constant increase in the total number of ED visits with PE diagnosis over time. During 2006–2008 there were 403,186 visits and 630,208 visits during 2016–2018 (*p* value < 0.0001). Concurrently, the rate of ED visits with PE per 100,000 US persons also rose from 42.1 in 2006–2008 to 64.2 in 2016–2018 (*p* value < 0.0001).

The percentage of male patients slightly increased from 44.9% to 47.7% (*p* value < 0.0001), the proportion of uninsured patients experienced a gradual decline from 5% to 4.7%, and the percentage of Medicaid-insured patients increased considerably from 8.2% to 12.7% (*p* value < 0.0001). On the other hand, there was an increase in the percentage of visits by patients residing in rural areas and those with low income (*p* value < 0.0001). Additionally, the proportion of patients discharged from the ED increased significantly from 7.5% to 24% (*p* value < 0.0001).

### 3.2. Trends in Hospitalization and Associated Costs

In 2016–2018, there was a decrease in the number of admitted patients to 478,958 patients (75.9%) when compared to 372,826 patients (92.4%) in 2006–2008 (*p* value < 0.0001), with an average age of 62.5 and 62.4, respectively. At the same time, there was a significant decline in the average LOS from 5.9 days in 2006–2008 to 4.2 days in 2016–2018 (*p* value < 0.0001). The mortality rate during hospital stays showed a gradual decline, from 3.4% in 2006–2008 to 2.8% in 2016–2018 (*p* value < 0.0001). However, the mean cost per admitted patient increased from $32,794 in 2006–2008 to $47,344 in 2016–2018 (*p* value < 0.0001). As well as aggregate cost, from $12,219,131,520 in 2006–2008 to $22,642,348,837 in 2016–2018 (*p* value < 0.0001).

## 4. Discussion

For the 12 years from 2006 to 2018, there was a steady increase in the rate of PE ED visits along with its associated impact. This finding mirrors the overall increase in US visit rates to the ED in general [11]. This observation can also be attributed to several factors, one of which is the introduction of multidetector CTPA, which has significantly improved the sensitivity and specificity for detecting PE, leading to its increased utilization and detection of more cases, including previously undiagnosed cases [4]. While these developments have enhanced the detection of PE, they also raise considerations of potentially over diagnosing subclinical cases as acute, which could lead to unnecessary treatment and increased healthcare costs [12]. Additionally, the period has seen an increase in the prevalence of some risk factors for PE, including immobility, medical comorbidities, obesity, and sedentary lifestyle [13].

At the end of the study period, there was a significant increase in the percentage of insured patients, mainly in the Medicaid subgroup, more noticeably in 2012–2014 which may represent the peak of the transformation of medical insurance status mix in the US. In concordance with the shrinkage of privately insured and uninsured patients, this is likely secondary to the implementation and adoption of the Affordable Care Act (ACA), commonly known as Obamacare. ACA aims to increase health insurance quality and accessibility, lower the uninsured percentage by expanding insurance coverage, and reduce costs of care [14].

In medical practice, every institution aims to decrease hospital LOS and increase discharge rates. The observed decrease in LOS and increase in discharge rate during the study period can be attributed to the introduction of NOACs, also known as Direct Oral Anticoagulants (DOACs). NOACs offer a rapid onset of action and have good safety profiles, enabling safer and earlier discharge of patients from the ED. Moreover, the shift towards outpatient management of PE, supported by clinical guidelines endorsing NOACs, has further supported these trends [15, 16]. Additionally, promoting early mobilization and implementation of optimized recovery protocols have collectively contributed to a more efficient and patient-centered approach to PE management. This multifactorial shift not only reflects advancements in medical treatment, but also aligns with broader objectives to optimize healthcare delivery and patient outcomes [17].

While the overall trends in patient outcomes show improvements in LOS and lower admission rates attributed to more discharge of PE patient from the ED due to the introduction of NOAC medications and follow up appointment arrangement. However, it is crucial to address the rising mean cost per admitted patient over the years. The cost increased significantly, from $32,794 in 2006–2008 to $47,344 in 2016–2018. This rise in costs could be attributed to various factors, such as increasing healthcare expenses in general, and advancements in medical technologies and its increased use [18]. It is probable that the increase in both cost per visit and diagnosis rates of PE contributed to the overall increase we noticed, even though there was a decrease in both LOS and admission rates. It is essential for healthcare systems and policymakers to closely monitor these costs and find strategies to ensure the affordability and accessibility of care for patients with PE (see Tables 1 and 2).

## 5. Limitation

There are a few constraints to consider in our study. The data available through HCUP is provided as aggregate, de-identified data, limiting the analysis at the individual patient level. Consequently, we were unable to evaluate the influence of comorbidities or the effects of multiple variables. Another limitation of this study is the exclusion of the transitional year 2015 due to the implementation of the ICD-10-CM coding system, replacing the older ICD-9-CM. This exclusion demonstrated a gap in data and potential variations in coding or reporting practices. Finally, Population estimates used for weighting may not perfectly align with census data, potentially affecting trend precision.

## 6. Conclusion

In conclusion, the trends in this study showed an increase in the rate of PE ED visits from 2006 to 2018, along with some positive trends, such as a decrease in admission rates, LOS, and mortality rates. However, there was an increase in the cost per admission and the overall aggregate cost, which presents a significant challenge. Further investigation is needed to ensure the sustainability of healthcare delivery for PE management.

## Figures and Tables

**Table 1:** Demographics of pulmonary embolism patients in US EDs from 2006 to 2018.

	2006–2008			2009–2011			2012–2014			2016–2018			*p* value⁣^∗^
	2006	2007	2008	2009	2010	2011	2012	2013	2014	2016	2017	2018	
No. of visits													
Accumulative	403,186			481,184			527,948			630,208			< 0.0001
Yearly	130,306	132,595	140,285	150,998	161,025	169,161	171,340	174,352	182,256	193,619	217,419	219,170	
Rate of visits per 100,000 US persons													
Accumulative	42.1			50.3			55.6			64.2			< 0.0001
Yearly	40.9	41.6	44	47.4	50.5	53.1	54.6	55.2	57.2	59.9	66.7	66.2	

Patient's characteristic													
Age mean (years)	61.9			61.5			61.3			61.1			0.287
Male gender (%)	44.9			46			47.1			47.7			< 0.0001
Payer													
Uninsured (%)	5			5.8			5.9			4.7			< 0.0001
Medicare (%)	50			48.8			49.3			48.9			
Medicaid (%)	8.2			6.7			11.3			12.7			
Private (%)	33.3			32.7			30.2			30.9			
Missed (%)	3.5			6			3.3			2.8			
Rural residence location (%)	16.9			17.6			17.6			18.1			< 0.0001
Low income for zip code (%)	25			24.9			27.4			27.7			< 0.0001
Discharged from ED (%)	7.5			9.4			13			24			< 0.0001

Hospital\Facility's characteristic													
North region of the US (%)	18.8			17.2			17.3			17.5			< 0.0001
Governmental ownership hospital (%)	12.5			11.4			10.9			13			< 0.0001
Rural location hospital (%)	13.9			14.1			13.8			13.7			0.4079
Teaching hospital (%)	42.6			42.4			51.4			63.6			< 0.0001
Trauma center hospital (%)	34.8			51.1			59.9			47.8			< 0.0001

*Note:* Table shows descriptive analysis of the demographics of pulmonary embolism patients in US EDs from 2006 to 2018 and the *p* value. *p* value calculated for each year separately (not as a group).

Abbreviations: ED: emergency department, US: United State.

⁣^∗^Compares the 2006 value with the 2018 value.

**Table 2:** Trends in US EDs for admitted pulmonary embolism patients from 2006 to 2018.

	2006–2008	2009–2011	2012–2014	2016–2018	*p* value⁣^∗^
No. of admitted to hospital (%)	372,826 (92.4%)	437,877 (90.5%)	459,315 (86.9%)	478,958 (75.9%)	< 0.0001
Age (mean)	62.4	62.1	62.1	62.5	0.154
Length of stay (mean days)	5.9	5.5	5	4.2	< 0.0001
Died during hospital stay (%)	3.4	3.1	2.9	2.8	< 0.0001
Discharge (from ward) (%)	61.2	62.9	64.5	66	< 0.0001
Costs, $ (mean)	32,794	36,870	41,288	47,344	< 0.0001
Aggregate costs, $	12,219,131,520	16,046,929,888	18,922,825,671	22,642,348,837	< 0.0001

*Note:* Table shows the descriptive analysis of the trends in US EDs for admitted pulmonary embolism patients from 2006 to 2018 and the *p* value. *p* value calculated for each year separately (not as a group).

Abbreviation: ED, emergency department.

⁣^∗^Compares the 2006 value with the 2018 value.

## Data Availability

Data are available online as part of The Healthcare Cost and Utilization Project at (https://www.hcup-us.ahrq.gov) by The Agency for Healthcare Research and Quality's. In addition, the raw data are available upon request and contact with the corresponding author.
